# Warm-up music and low-dose caffeine enhance the activity profile and psychophysiological responses during simulated combat in female taekwondo athletes

**DOI:** 10.1038/s41598-024-64880-1

**Published:** 2024-06-21

**Authors:** Slaheddine Delleli, Ibrahim Ouergui, Hamdi Messaoudi, Craig Bridge, Luca Paolo Ardigò, Hamdi Chtourou

**Affiliations:** 1 High Institute of Sport and Physical Education of Sfax, University of Sfax, Sfax, Tunisia; 2 Research Unit: Physical Activity, Sport and Health, UR18JS01, National Observatory of Sport, 1003 Tunis, Tunisia; 3 High Institute of Sport and Physical Education of Kef, University of Jendouba, 7100 El Kef, Tunisia; 4 Research Unit: Sports Science, Health and Movement, UR22JS01, University of Jendouba, 7100 El Kef, Tunisia; 5 Sports Performance Research Group, Edge Hill University, Wilson Centre, Ormskirk, UK; 6 Department of Teacher Education, NLA University College, Linstows Gate 3, 0166 Oslo, Norway

**Keywords:** Ergogenic aids, Activity profile, Attack, Performance, Physiology, Psychology

## Abstract

To assess the effects of warm-up music and low dose (3 mg·kg^−1^) of caffeine (CAF) on female taekwondo athlete’s activity profile and psychophysiological responses during simulated combat. In a double-blinded, randomized, crossover study, 16 female athletes participated in simulated combats under one control and 5 experimental conditions [i.e., CAF alone (CAF), placebo alone (PL), CAF with music (CAF + M), PL with music (PL + M), and no supplement with music (M)]. After warming-up, athletes rated their felt arousal (FAS). Mean (HR_mean_) and peak (HR_peak_) heart rate values were determined for each combat. After fighting, athletes rated their perceived exertion (RPE), feeling scale (FS), FAS, and physical enjoyment (PACES). Time-motion and technical-tactical variables were analyzed. CAF + M induced shorter skip and pause time, while attack time increased compared to other conditions (*p* < 0.05). Moreover, CAF + M increased single attacks, combined attacks, counter-attacks (*p* < 0.001), and defensive actions (*p* < 0.05) than other conditions. HR_mean_ and HR_peak_ were lower under CAF + M than other conditions (*p* < 0.05). Additionally, higher FAS post-combat, FS, and PACES were observed under CAF + M, while RPE was lower (except CAF condition) compared to the other conditions (*p* < 0.05.Using CAF with warm-up music may increase combat cadence and improve the psychological state in female athletes more effectively than either strategy alone.

## Introduction

In combat sports, such as taekwondo, competitions are organized according to weight categories, competition level, and sex^1^. During taekwondo combat, two opponents from the same category fight across three 2-min rounds (1 min rest in-between) with the objective of overcoming an opponent by obtaining a greater quantity of points for the execution of techniques to permitted scoring areas or by achieving a technical knockout^2^. Success within the sport may be determined by athletes’ technical, tactical, psychological, physical, and physiological characteristics^1^. The chances of winning an elite championship competition are marginal and require athletes to be able to effectively manage the anticipatory stress-response, changeable physiological responses, and potential fatigue that may arise from both single and repeated combat exposures^3,4^.

In the quest for optimal performance, athletes and coaches have a range of potential pre-conditioning and nutritional strategies at their disposal, many of which can be employed to facilitate performance on the day of competition^5,6^. In the context of the latter, nutritional ergogenic aids, such as caffeine (CAF), have the potential to acutely facilitate performance in a variety of settings and hence they are widely consumed by athletes^6,7^. Performance improvements following ingestion of CAF may be manifested through a variety of potential mechanisms, including binding to adenosine receptor sites, concomitant release of neurotransmitters, altered RPE and pain perception, enhanced muscle contractile properties, and improved intramuscular metabolic environment^8,9^.

Despite compelling evidence to support the efficacy of CAF in a variety of sport settings in males, research investigating the efficacy of CAF in females is in its relative infancy. Caution should remain in considering males and females as a homogenous group due to the observed differences in physiology and hormones that could potentially influence processes of absorption and metabolism^6,10^. Indeed, there is evidence to suggest differences in the ergogenic effect of CAF on performance across different sports^11^ and the occurrence of negative psychological disposition^12^ between males and females, albeit this has not been observed consistently^13,14^. Although the precise process causing the various reactions to the supplement is not yet understood, body composition and size may be among the influencing factors^12^. Specifically, CAF is mainly distributed through free body mass^15^, The larger percentage of adipose tissue may lead to higher CAF concentrations in the tissue and plasma^12^. Since female athletes tend to demonstrate greater body fat than their male counterparts^1^, this will also influence CAF absorption, plasma concentrations, and the occurrence of side effects^16^. Additionally, steroid hormone levels’ variation in females could influence the reactions brought on by CAF^17^. Actually, changes in hormone levels throughout the menstrual cycle affect the rate at which CAF is metabolized as well as the neuromuscular system function^17^. Concerted efforts have recently been made to address such deficiencies, and evidence is indeed emerging to support CAF’s ergogenic potential in female athletes in various sports settings^6,18,19^. Recent research in taekwondo has identified performance improvements on specific taekwondo tests following ingestion of 3 mg·kg^−1^ body mass of CAF in male or mixed samples of athletes^20^, albeit the translation of these findings to actual performance in combat is debatable. A limited number of studies have investigated the influence of CAF on performance in simulated combat using males or with unreported genders, with conflicting findings in terms of efficacy^21,22^. Further research investigating the influence of CAF on the activity profiles and psychophysiological responses to combat using female athletes is warranted.

Recent investigations using males^23^ or a mixed sample of taekwondo athletes^20^ have reported that combining CAF with other ergogenic aids or other warm-up strategies can result in additive effects on performance beyond that seen when using either strategy in isolation. Since it serves as a psychological ergogenic aid, music has been reported to improve affective balance, behavior, and cognitive skills associated with physical performance enhancement^24,25^. Interestingly, applying musical stimulus prior to exercise has received significant interest in the field of music research^26^. This is largely a function of the restrictions imposed on the use of audio devices during competition in different sports, and more specifically in taekwondo contests^27^. In this regard, warm-up music can be effective in delaying fatigue, and improving affective state associated with physical performance enhancement, especially, when the stimulus is self-selected^26^. However, such findings cannot be generalized between sexes, since the responsiveness to warm-up music intervention is often greater within male compared with female athletes^27,28^.

Despite the endocrinological differences between the sexes and the growing interest in menstrual cycle-specific research, exercise science and nutrition research continue to generalize results between males and females^29^. Given the significant differences in biology and behavior between genders, such inferences are not acceptable^30^. Whilst CAF^18^ and warm-up music^26^ benefits on female athletes have received only limited research attention, their synergistic effects have not been studied. Therefore, the present study investigated the acute effects of low dose of CAF and preferred warm-up music on the subsequent female taekwondo athletes’ responses during simulated combats. It has been documented that CAF acts as an adenosine antagonist by blocking its A_1_ and A_2a_ receptors leading to improved neurotransmission, muscular excitation–contraction coupling, and sympathetic nervous system’ s activation while reducing pain perception^9,31,32^. Differently, improvements in performance following music listening are mediated through improved mood, exercise enjoyment, motivation and increased feelings of power, while reduced fatigue symptoms^24,27^. Since success in taekwondo competition is multifactorial and may be determined by the collective interplay between psychological responses, physical abilities and technical-tactical skills^1^, it was hypothesized that combining CAF and warm-up music may increase females athletes activity profiles and elicit more desirable psycho-physiological responses when compared with using either strategy in isolation.

## Material and methods

### Participants

Female athletes were recruited following a convenience sampling, as long as they met the following inclusion criteria: (a) classified as an active elite female athlete; (b) do not suffer from any restrictions to sports practice or hearing loss; (c) minimum age of 17 years with at least 7 years of taekwondo experience; (d) were not currently taking any form of contraceptives as its use may interferes with CAF metabolism and excretion^14^, and (e) have a regular menstrual cycle, defined as a variation lower than 3 days in the range of their menstrual cycles' length^14^ for the previous 2 months. The sample size required was priori estimated using the G*Power software (Version 3.1.9.4, University of Kiel, Kiel, Germany). The repeated measures ANOVA, within factors test, with six conditions revealed that a total sample size of 14 would be sufficient to find medium significant effects of condition (effect size f = 0.30, α = 0.05) with an actual power of 83%. Taking into consideration the risk of athlete dropout, 16 female elite black belt taekwondo athletes (age: 17 to 19 years; body mass: 43 to 59 kg; height: 152 to187 cm) were eligible and volunteered to participate in the present study. Athletes were distributed into < 49 kg (8 athletes), < 57 kg (6 athletes) and < 67kg (2 athletes) weight categories. They were training regularly 5 sessions per week, with each session lasting 2 h. According to the questionnaire of Bühler et al.^33^, the mean habitual CAF consumption ranged from 0.5 to 2.1 mg^.^kg^−1^ of body mass, indicating that athletes were low CAF consumers. Athletes reported an average menstrual cycle duration of 28 ± 1 day. The recruited athletes had no more than two days difference in-between even in the follicular (8 athletes) or luteal phase (8 athletes). The participants were asked to follow the same diet, avoid alcoholic substances and vigorous exercise, and restrain from CAF consumption (in drinks and supplements) 48 h before each experimental session. All participants and their parents were informed about the procedures, the possible risks and side effects involved in the investigation and they signed a written informed consent form. This study was conducted in accordance with the last Declaration of Helsinki and the protocol was fully approved by the Committee of protection of southern persons (CPP SUD N◦ 0332/2021) before commencing experimentation.

### Experimental design

Before commencing experimentation, athletes were familiarized with the testing procedures and questionnaires, and each athlete was instructed to select their most preferred music (i.e., music with tempo > 120 bpm) and indicate its motivational quotient using the Brunel Music Rating Inventory-2^34^. In the testing sessions, athletes performed simulated combats using a double-blind, counterbalanced, crossover study design. Specifically, athletes ingested 3 mg^.^kg^−1^ of CAF or placebo (PL) (i.e., 3 mg^.^kg^−1^ of placebo comprising all-purpose bleached flour), in a double blind fashion. Allocation and assignment for each participant was performed by an independent, blinded staff member of our research team. During supplementation, participants were instructed to not discuss exchange or compare tastes and the investigators of the studied athletes supervised consumption compliance. Each trial was given an alphanumeric code in order to keep participants and researchers unaware of the supplement being tested in each experiment. This code was disclosed following the variables' analysis^35^. Following the supplements consumption, verbal questioning demonstrated that almost all of the participants were unable to differentiate between the supplements, proving the efficacy of blinding. After supplementation, athletes remained seated for 50 min and then executed an 8-min warm-up (i.e., included 4 min of low-intensity running and 4 min of technical drills) either with or without listening to their preferred music. The priori selected music was intensive with tempos ranging from 124 to 168 bpm. For each athlete, one preferred music was used for all music conditions and music volume was fixed at 80 dB for all participants^28^. The music track was looped in cased it ended before the warm-up was completed. In the no music conditions, headphones were worn but no music was played. A two-min rest period was given after the warm–up and the combats were subsequently conducted. Consequently, the fights were performed under five experimental and a control condition including: (a) no supplement without music (control); (b) CAF without music (CAF); (c) PL without music (PL); (d) CAF with music (CAF + M); (e) PL with music (PL + M) and (f) no supplement with music (M). Athletes were asked to rate their perceived activation after warming-up and immediately after fighting using the felt arousal scale (FAS), while feeling scale (FS), physical activity enjoyment (PACES) and the rating of perceived exertion (RPE) were determined after the combats. During each fight, mean and peak heart rate (HR_mean_ and HR_peak_) were determined. For each testing session, participants had their pre-competition meal (three hours before the onset of the trial) and 500 ml of water (two hours before the onset of the trial). To provide enough recovery time between interventions and ensure CAF removal, 7-day of washout period between the sessions were guaranteed^36^. All the sessions were scheduled in the evening hours (17:00–18:00 p.m) within their regular training timing sessions (i.e., 17:00–19:00 p.m).

### Testing procedures

#### Simulated combats

The simulated combats were performed in accordance with official rules of World Taekwondo following the Olympic weight categories^2^. The combats consisted of three 2-min rounds interspersed with 1-min rest interval in which athletes wore their official protective equipment. A black belt taekwondo coach who was blinded to the supplement and the musical intervention that the athletes had received refereed the matches. All combats involved partners from the same condition (e.g., CAF vs. CAF)^21^. Block randomization method was used using an excel spreadsheet (Microsoft Excel 2007, Redmond, WA, USA) to arrange the combats and ensure a balance in sample size. All combats were recorded for subsequent analysis using 2 cameras (Canon 650D 18 Megapixels, ISO: 400, shutter speed: 1/125 s, f/4; Canon, Inc., Tokyo, Japan) placed at 1.5 m from the combat area.

#### Videos-analysis

The technical-tactical analysis included the determination of single attacks, combined attacks, counterattacks, and defensives actions. The time-motion variables included the attacks time, skip time and pause time. The recorded video footage was analyzed frame-by-frame using the Kinovea software (V 0.9.5, http://www.kinovea.org/). Two investigators who were highly experienced and familiar with taekwondo matches analyzed the combats and repeated the process one week later. Consensus was used to resolve disagreements between the two researchers. The values of Cohen’s kappa coefficient for inter-observer agreement and intraclass correlation coefficient for intra-observer agreement were higher than 0.9 indicating a high reliability of the video-analysis system.

#### Heart rate measurements

Heart rate (HR) was measured every 5 s throughout the taekwondo combats (Polar Team^2^ Pro System, Polar Electro OY, Kempele, Finland) and mean (HR_mean_) and peak (HR_peak_) values were used for the analysis.

#### Rating of perceived exertion

Perceived exertion was assessed using the CR-10 Borg scale^37^. This is a scale ranging from “0” to “10”, with corresponding verbal expressions, gradually increase with the perceived sensation’s intensity (0 = nothing at all; 1 = Very week; 2 = week; 3–4 = Moderate; 5–6 = strong; 7–9 = very strong; and 10 = extremely strong). The Cronbach’s alpha of the scale in the present study was 0.75.

#### The physical activity enjoyment scale (PACES)

The18 items original scale version was used to detect the level of pleasure and enjoyment of participants^38^. Items involved 11 negative and 7 positive items measured through a 7-point score ranging from 1 to 7^38^ with the score (*i.e.,* the sum of total responses for each athlete) range from 18 to 126. The Cronbach’s alpha of the scale in the present study was 0.79.

#### Feeling scale (FS)

The FS utilizes a single-item 11-point bipolar rating scale ranging from − 5 to + 5, with the stem ‘‘How do you currently feel?’’. Anchors are given at 0 (Neutral) and all odd integers, ranging from ‘‘Very Bad’’ at − 5 to ‘‘Very Good’’ at + 5^39^. The Cronbach’s alpha of the scale was 0.84 for the present study.

#### Felt arousal scale (FAS)

The FAS was used to measure arousal along a 6 points scale ranging from low arousal (1 point) to high arousal (6 points)^40^. The participants were instructed to mark the scale on the basis of their perceived activation after warming-up and fighting. The Cronbach’s alpha of the scale was 0.72 for the present study.

### Statistical analysis

The statistical analysis was performed using SPSS 20.0 statistical software (IBM corps., Armonk, NY, USA). Data were presented as mean and standard deviation, and Median and Interquartile range values were reported for non-normal distribution data. The Shapiro–Wilk test was used to check and confirm the normality of data sets, and the Levene test was used to verify the homogeneity of variances. Sphericity was tested using the Mauchly test. For variables with normal distribution, a one-way analysis of variance (ANOVA) (condition) with repeated measurements was used, with Bonferroni adjustment for post hoc comparisons. Standardized effect size analysis (Cohen’s d) was used to interpret the magnitude of differences between variables and considered as: trivial (≤ 0.20); small (≤ 0.60); moderate (≤ 1.20); large (≤ 2.0); very large (≤ 4.0) (very large); and extremely large (> 4.0)^41^. The non-parametric Friedman test was used with the Wilcoxon signed rank test as post hoc for those variables that were not normally distributed. The rank biserial correlation coefficient (r) was calculated using the Wilcoxon Z-scores and the total number of observations (N) (i.e., r = Z/√N) and considered as 0.1 to < 0.3 (small), 0.3 to < 0.5 (moderate) and ≥ 0.5 (large) ^42^ The level of statistical significance was set at *p* ≤ 0.05.

## Results

### Time-motion variables

For attack time, there was a main effect of condition (Chi^2^ = 70; df = 5; N = 16; *p* < 0.001), with CAF + M resulting in longer time than the other conditions (all, Z = − 3.52; r = 0.88; *p* < 0.001). Similarly, for skipping time, there was a main effect of condition (F_5,11_ = 245.50; *p* < 0.001; η_p_^2^ = 0.99), with CAF + M eliciting shorter skipping time than the other conditions (all *p* < 0.001). Additionally, there was a main effect of condition for pause time (Chi^2^ = 36.61; df = 5; N = 16; *p* < 0.001), with CAF + M resulting in shorter time than control (Z = − 3.37; r = 0.84; *p* = 0.001), as well as M, CAF, PL and M + PL (all, Z = − 3.52; r = 0.88; *p* < 0.001) (Table 1).

**Table 1:** Time-motion variables during simulated combat following different conditions (N = 16).

	Control		M		CAF		PL		PL + M		CAF + M	
	Mean (SD)	Med/IQR	Mean (SD)	Med/IQR	Mean (SD)	Med/IQR	Mean (SD)	Med/IQR	Mean (SD)	Med/IQR	Mean (SD)	Med/IQR
Attack time (s)	–	74.62/12.38	–	90.25/8.58^a,d^	–	91.77/2.63 ^a,d^	–	73.71/9.82	–	91/6.13^a,d^	–	125.78/4.63^a,b,c,d,e^
Skip time (s)	235.41 (8.80)	–	218.79 (7.99)^a,d^	–	219.74 (5.75)^ad^	–	235.01 (6.92)	–	221.48 (5.56)^ad^	–	193.42 (5.11)^a,b,c,d,e^	–
Pause time (s)	–	51.17/11.78	–	50.48/8.49	–	47.98/6.40*^,$,ɸ^	–	50.67/3.92	–	47.22/4.38^$,ɸ^	–	40.50/4.57*^,b,c,d,e^

^a^different from control at *p* < 0.001; ^b^different from M at *p* < 0.001; ^c^different from CAF at *p* < 0.001; ^d^different from PL at *p* < 0.001; ^e^different from PL + M at *p* < 0.001; *different from control at *p* < 0.05; ^$^different from M at *p* < 0.05; ^ɸ^different from PL at *p* < 0.05; M: music; CAF: caffeine; PL: placebo; PL + M: placebo and music; CAF + M: caffeine and music; SD: standard deviation; Med/IQR: median/interquartile range; s: second.

### Technical-tactical aspects

**Figure 1:**
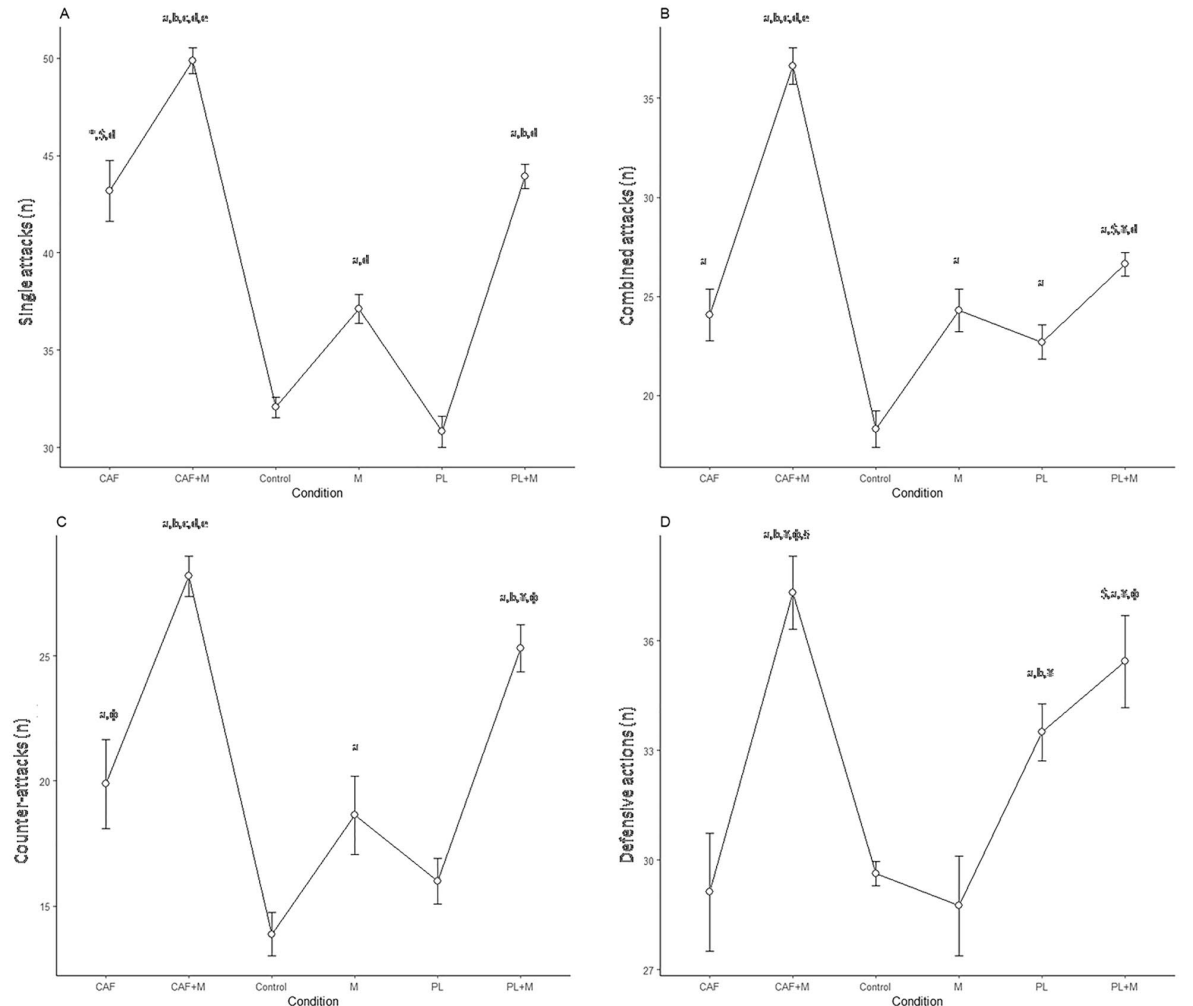
Technical-tactical performance during simulated combat under different conditions (N = 16). Values are presented as mean (circle) and confidence interval (vertical line) with line plot; (**A**) single attacks; (**B**) combined attacks; (**C**) counter-attacks; (**D**) defensive actions; a: different from control at *p* < 0.001; b: different from M at *p* < 0.001; c: different from CAF at *p* < 0.001; d: different from PL at *p* < 0.001; e: different from PL + M at *p* < 0.001; *: different from control at *p* < 0.05; $: different from M at *p* < 0.05; ɸ: different from PL at *p* < 0.05; ¥: different from CAF at *p* < 0.05; §: different from PL + M at *p* < 0.05; M: music; CAF: caffeine; PL: placebo; PL + M: placebo and music; CAF + M: caffeine and music; n: number.

There was a main effect of condition for single attacks (Chi^2^ = 75.78; df = 5; N = 16; *p* < 0.001), with CAF + M resulting in higher attacks than control, PL and CAF (Z = − 3.53; r = 0.88; *p* < 0.001), as well as M and PL + M (Z = − 3.54; r = 0.89; *p* < 0.001). In addition, for combined attacks, there was a main effect of condition (F_5,11_ = 337; *p* < 0.001;η_p_^2^ = 0.99), with CAF + M eliciting higher attacks than the other conditions (all, *p* < 0.001). Furthermore, for counter-attacks, there was a main effect of condition (F_5,11_ = 85.88; *p* < 0.001;η_p_^2^ = 0.98), with CAF + M eliciting higher number of counter-attacks than the other conditions (all *p* < 0.001). Finally, there was a main effect of condition for defensive actions (Chi^2^ = 62.87; df = 5; N = 16; *p* < 0.001), with CAF + M resulting in higher actions than control, M (both, Z = -3.53; r = 0.88; *p* < 0.001), CAF (Z = − 3.42; r = 0.86; *p* = 0.001), PL (Z = − 3.40; r = 0.85; *p* = 0.001), and PL + M (Z = − 2.68; r = 0.67; *p* = 0.007) (Fig. 1).

### Heart rate responses

For HR_mean_, there was a main effect of condition (F_5,11_ = 29.31; *p* < 0.001;η_p_^2^ = 0.93), with CAF + M eliciting lower values compared with the other conditions (all *p* < 0.001). Similarly, there was a main effect of condition for HR_peak_ (Chi^2^ = 48.62; df = 5; N = 16; *p* < 0.001), with CAF + M eliciting lower values than control (Z = − 3.36; r = 0.84; *p* = 0.001), M (Z = − 3.21; r = 0.80; *p* = 0.001), CAF (Z = − 3.44; r = 0.86; *p* = 0.001), PL (Z = − 3.53; r = 0.88; *p* < 0.001), and PL + M (Z = − 3.52; r = 0.88; *p* < * p* < 0.001) (Table 2).

**Table 2:** Perceived exertion and heart rate responses in simulated combat following different conditions (N = 16).

	Control		M		CAF		PL		PL + M		CAF + M	
	Mean (SD)	Med/IQR	Mean (SD)	Med/IQR	Mean (SD)	Med/IQR	Mean (SD)	Med/IQR	Mean (SD)	Med/IQR	Mean (SD)	Med/IQR
HR _peak_ (beats/min)	–	198/2^¥^	–	197/2.75^¥,^*	–	201/2.5	–	196.5/2.5^¥^	–	196.5/3.25^¥^		187.5/5.75*^,¥,$,d,e^
HR _mean_ (beats/min)	156.81 (2.76)	–	155.38 (3.88)^¥^	–	158.88 (4.81)	–	157.38 (1.50)	–	155.19 (2.83)	–	148.56 (2.73)^a,b,c,d,e^	–
RPE (a.u)	–	9/1.5	–	9/0.75	–	8/1*^,$,ɸ,§^	–	9/0.75	–	9/0.75	–	8/1*^,$,ɸ,§^

^a^different from control at *p* < 0.001; ^b^different from M at *p* < 0.001; ^c^different from CAF at *p* < 0.001; ^d^different from PL at *p* < 0.001; ^e^different from PL + M at *p* < 0.001; *different from control at *p* < 0.05; ^$^different from M at *p* < 0.05; ^ɸ^different from PL at *p* < 0.05; ^¥^different from CAF at *p* < 0.05; ^§^different from PL + M at *p* < 0.05; M: music; CAF: caffeine; PL: placebo; PL + M: placebo and music; CAF + M: caffeine and music; SD: standard deviation; Med/IQR: median/interquartile range; HR_peak_: heart rate peak; HR_mean_: heart rate mean; batt/min: battements per minute; RPE: perceived exertion; a.u: arbitrary unit.

### Perceived exertion

There was a main effect of condition (Chi^2^ = 23.40; df = 5; N = 16; *p* < 0.001), with CAF + M eliciting lower values than control (Z = − 2.51; r = 0.63; *p* = 0.012), M (Z = − 2.84; r = 0.71; *p* = 0.005), PL (Z = − 2.68; r = 0.67; *p* = 0.007), and PL + M (Z = -3.07; r = 0.77; *p* = 0.002) (Table 2).

### Feeling scale

There was a main effect of condition (Chi^2^ = 48.22; df = 5; N = 16; *p* < 0.001), with CAF + M eliciting higher values than control (Z = − 3.53; r = 0.88; *p* < 0.001), M (Z = − 3.33; r = 0.83; *p* = 0.001), CAF (Z = − 3.57; r = 0.89; *p* < 0.001), PL (Z = − 3.53; r = 0.88; *p* = 0.001) and PL + M (Z = − 3.48; r = 0.87; *p* = 0.001) (Table 3).

**Table 3:** Female athletes’ psychological responses to each condition (N = 16).

	Control		M		CAF		PL		PL + M		CAF + M	
	Mean (SD)	Med/IQR	Mean (SD)	Med/IQR	Mean (SD)	Med/IQR	Mean (SD)	Med/IQR	Mean (SD)	Med/IQR	Mean (SD)	Med/IQR
FAS post warm-up	–	1.5/1	–	3/1*^¥ɸ§^	–	2/1	–	2/1	–	1.5/1	–	2/1*^ɸ§^
FAS post combat	–	2/1	–	2.5/1*^ɸ^	–	3/2*^ɸ^	–	2/1.75	–	3/1*^ɸ^	–	5/1.75^a$¥d§^
FS	–	0/2	–	1.5/1*^ɸ^	–	1/0.75*	–	0/1	–	1/0.75*	–	4/1.75^a$cɸ§^
PACES	62.63 (4.40)^d^	–	59.94 (4.25)^d^	–	63.56 (4.32)^d^	–	53.44 (2.28)	–	58.75 (5.45)^ɸ^	–	69.19 (3.37)*^,b,c,d,e^	–

^a^different from control at *p* < 0.001; ^b^different from M at *p* < 0.001; ^c^different from CAF at *p* < 0.001; ^d^different from PL at *p* < 0.001; ^e^different from PL + M at *p* < 0.001; *different from control at *p* < 0.05; ^$^different from M at *p* < 0.05; ^ɸ^different from PL at *p* < 0.05; ^¥^different from CAF at *p* < 0.05; ^§^different from PL + M at *p* < 0.05; M: music; CAF: caffeine; PL: placebo; PL + M: placebo and music; CAF + M: caffeine and music; SD: standard deviation; Med/IQR: median/interquartile range; FAS: felt arousal scale; FS: feeling scale; PACES: physical activity enjoyment scale.

### Felt arousal after warm-up

There was a main effect of condition (Chi^2^ = 19.27; df = 5; N = 16; *p* = 0.002), with CAF + M eliciting higher values than control (Z = -2.30; r = 0.58; *p* = 0.022), PL (Z = − 2.07; r = 0.52; *p* = 0.039), and PL + M (Z = − 2.14; r = 0.54; *p* = 0.033) (Table 3).

### Felt arousal post_combat

There was a main effect of condition (Chi^2^ = 46.86; df = 5; N = 16; *p* < 0.001), with CAF + M eliciting higher values than control (Z = − 3.55; r = 0.89; *p* < 0.001), M (Z = − 3.40; r = 0.85; *p* = 0.001), CAF (Z = − 3.25; r = 0.81; *p* = 0.001), PL (Z = − 3.55; r = 0.89; *p* < 0.001), and PL + M (Z = − 3.32; r = 0.83; *p* = 0.001) (Table 3).

### Physical enjoyment

There was a main effect of condition (F_5,11_ = 42.65; *p* < 0.001; η_p_^2^ = 0.95), with CAF + M eliciting higher values compared with control (*p* = 0.001) and the other conditions (all *p* < 0.01(Table 3).

## Discussion

This study provides novel insights into the effects of warm-up music and low dose CAF intake on female athletes’ performances during simulated taekwondo combat. As there are limited available data on the effects of these ergogenic aids in competitive events, especially within female athletes, this study is the first to address the synergetic effects of warm-up music and low dose of CAF on technical-tactical skills, time-motion variables and psycho-physiological responses during simulated combats. The overarching findings of the study suggest that combining a low dose of CAF with warm-up music could be a more effective strategy to increase combat cadence and generate more desirable psychological state in female athletes when compared with using either strategy alone.

The increase in the number of offensive and defensive actions performed under CAF + M condition suggest an increase in the intensity of combats through promoting more dynamic competition routine. It was previously reported that a moderate dose of CAF (i.e., 5 mg·kg^−1^) improved the total number of attacks performed in the second combat by 37.39% in male taekwondo athletes^21^. However, female athletes’ responses to warm-up music and CAF interventions during a competitive event were not investigated, meaning that it is difficult to form definitive comparisons between the studies. It is important to realize that maximal performance is associated with the execution of repeated high-intensity techniques during the entire match^43^. The ability to maintain intensive actions requires the development of specific physical attributes which rely heavily on both anaerobic and aerobic metabolism^1^. Using a similar CAF dose (i.e., 3 mg·kg^−1^), but a mixed sample of athletes, Ouergui et al.^20^ reported that CAF ingestion elicited a higher number of kicks recorded during the frequency speed of kick test (FSKT) using both simple (i.e., 10 s) and multiple (i.e. 5 × 10 s) versions. Both versions replicate some important characteristics/actions typically observed during the match^44^. This finding was confirmed in a later study comparing CAF effects in relation to competitive level and sex^45^, where female elite athletes also benefited from low dose CAF intake during the aforementioned tests. Ouergui et al.^28^ also reported that taekwondo athletes benefit from listening to music, as they were able to perform a higher number of techniques’ on such tests, particularly when listening to preferred music. For the first time, the present study shows that the combined use of warm-up music and low dose CAF ingestion may have additive effects on female athletes performance in simulated combat, a setting which is arguably a more ecologically valid.

During a typical contest, combat sports’ athletes use a variety of offensive and defensive actions to overcome the opponent^46^. Opponent behavior can influence athletes’ technical-tactical skills^46^. For instance, delivering a higher number of offensive actions often forces the opponent to counterattack, and vice versa, which may increase the overall intensity of the fight^3^. In the present study, the combined intervention (i.e., CAF + M) resulted in the execution of a greater number of counterattacks and defensive actions compared with the other conditions. During official taekwondo combats, time-motion analysis revealed that winners tended to display higher anaerobic power and execute a higher number of techniques when compared with their defeated counterparts^47^. Interestingly, female winners were reported to deliver more techniques during the combat when compared with their successful male counterparts^48^, which may highlight the effectiveness of this combination in increasing female athletes’ chance to win a competition.

Amongst the antagonist actions of CAF on adenosine is the activation of the sympathetic nervous system^49^. However, CAF effect on cardiac outputs appears to be dose^18^ and exercise intensity dependent (i.e., decline as exercise intensity increases)^49^. In the context of music, music preference correlates positively with relaxation^50^, with recent evidence^51^ reporting that upbeat music produced feelings of relaxation in male athletes. In the present study, preceding warm-up music by supplementation with low dose of CAF resulted in lower peak and mean heart rate values. While no neurological or hormonal measurements were conducted to explain mechanisms behind this response, maintaining lower HR values may reflect quicker recovery rate during the low-intensity actions due to improved parasympathetic recovery^52^ and a reduction in the central fatigue rate^53^. This postulation may be indirectly supported by time-motion results, since combining CAF and warm-up music reduced the skipping time. The increased combat cadence may reflect an improved ability to accelerate and achieve the opponent in a short distance and time^54^. This is not surprising since increases in vigor and reaction time’ are common CAF^55^ and warm-up music^56^ actions. Additionally, a decrease in fatigue perception could be among the reasons to maintain high-intensity actions^31^, which may influence the combat rhythm. Based on the fact that RPE score correlates with HR values during taekwondo contest^57^, it is not surprising that RPE and HR values simultaneously were lower under CAF + M condition. A decrease in RPE may reflect the ability of mitigating afferent nerves’ feedback, reducing the acute physiological responses^58^*.*

It has been reported that the ergogenic potential of CAF on taekwondo specific performance is dependent on competition level, with greater benefits in elite than sub-elite athletes^45^. For warm-up music, recent meta-analysis revealed that this stimulus present greater effects in highly trained subjects than less trained ones regarding psychological responses and the reverse for physical performances^26^. Since participants in the present study are elite athletes, their greater responses to the CAF + M condition may be related to complementary actions by CAF and music at the central and peripheral levels^23^. Specifically, CAF low doses (i.e., ≤ 3 mg^.^kg^−1^) act mainly on the central nervous system by increasing the prefrontal cortex activity and enhancing executive function^59^. For warm-up music, stimulus increased brain activity, which improved decision-making and preparation for action in sport^56^. At the peripheral levels, CAF^9^ and music^60^ enhance muscular contractility, through power production increase due to neuromuscular activity’s changes. This is supported by CAF enhancing effects of anabolic processes^22^ and music activating effects of muscles’ oxygenation^61^. From a psychological point of view, it is very interesting to highlight that CAF + M condition elicited better physical enjoyment and affective valence compared with their isolate use. A recent meta-analysis reported that affective valence scores were directed toward the positive end of the scale when pre-task music was used^26^. When considering FAS, warm-up music alone was sufficient to induce greater perceived activation before competing. However, after combat such effects were altered and higher values were recorded under CAF + M condition. This may indicate that the participants started the fight highly energized under music condition, but this effect diminished as the combat progressed^62^. Therefore, preceding warm-up music by low dose of CAF may extend its effects, may be via its analgesic potential and dopamine stimulation^7^.

In comparison to males, the complex nature of the scientific framework surrounding the menstrual cycle has been challenging, limiting the development and funding of numerous research studies^30^. However, CAF^13,14^ and warm–up music^63^ ergogenic potentials have been established through the menstrual cycle. Because the most common symptoms experienced during the menstrual cycle are mood changes/anxiety, and tiredness/fatigue^64^, such results may be reasonable since CAF and music commonly serve to regulate affective and neuromuscular responses^9,26,60^.

Although the present study provides unique and interesting findings to female athletes, some limitations should be declared. In fact, no neurological or hormonal measurements were conducted to explain the mechanisms under the synergetic effect of CAF and warm-up music. Additionally, the results are presented for the whole menstrual cycle and not according to different phases. Moreover, diet was not controlled during the whole study period and athletes were only asked to maintain their normal diet, which may implicate difference in macronutrient intake between subjects. Finally, for ecological validity matter, self-selected music was used without controlling the genre and synchronization with the performed warm-up.

## Conclusions

The present study showed that preceding warm-up music by low dose of CAF induced greater activity profile and more desirable psycho-physiological responses in female taekwondo athletes. In fact, the combine use of these strategies increased the combat intensity whist lowering athletes RPE and HR responses. In addition, CAF + M increased technical actions and guaranteed more desirable psychological responses. Since CAF was supplemented relatively to body mass and the music was preferred, the present study may suggest that using more personalized ergogenic strategies would be effective to cope with competition demands. Furthermore, although this study did not analyze the possible moderating effects of the menstrual cycle phases, benefits under CAF + M condition during the whole menstrual cycle indicated the robustness of their synergistic effects.

## Data Availability

All data generated and analyzed during this study are available from corresponding author.
